# Rapid on-site radionuclide screening of aqueous waste streams using dip-stick technologies and liquid scintillation counting

**DOI:** 10.1007/s10967-017-5413-9

**Published:** 2017-08-18

**Authors:** Phillip E. Warwick, Ian W. Croudace

**Affiliations:** GAU-Radioanalytical, University of Southampton, European Way, Southampton, SO14 3ZH UK

**Keywords:** ^90^Sr, ^99^Tc, Aqueous waste streams, Rapid screening, Dip-stick technology

## Abstract

This paper describes an early-stage evaluation of a purpose-designed extraction/detection system that can be deployed by non-specialists either on-site or as part of a mobile laboratory. The system comprises three main components; (1) an optimised compact extraction system for recovery of radionuclides from the waste form; (2) an extraction test strip designed to recover the radionuclides from the waste digest; (3) a scintillation-based detection system capable of quantification of alpha, low energy beta and high energy beta emitting radionuclides. Data are presented on the preliminary assessment of the extraction/detection system for the measurement of ^90^Sr and ^99^Tc in aqueous wastes.

## Introduction

Robust characterization of nuclear wastes is critical in underpinning nuclear decommissioning programmes and its importance has been recently highlighted by Emptage et al., [1]. Traditionally nuclear waste characterisation has relied on specialist laboratory radioanalytical support which, although providing high quality data, can result in delays to the decommissioning programme. Although such support is essential, many areas of waste characterization can be accelerated through careful integration of detailed laboratory characterization with quicker, less sensitive on-site screening capabilities. Screening can provide valuable information on the presence/absence of contamination, stability of the waste stream composition and radionuclide vectors and early indication of significant deviations from expected operational parameters. For example, the procedures have been developed for rapid screening of potable waters in the event of an intentional assault on the drinking water supply system [2]. On-site screening has been widely adopted for easy-to-measure (ETM; categorization [3]) gamma-emitting radionuclides. Development of on-site techniques for difficult-to-measure (DTM) radionuclides is desirable and techniques such as autoradiography have been applied to identify contaminated objects and surfaces (e.g., [4, 5]). However, extension of this concept to nuclide-specific measurement of radionuclides such as ^90^Sr and ^99^Tc has been limited, mainly as a result of the technical difficulties associated with the robust detection of these radionuclides outside of the laboratory environment.

Dip-stick technology applied to rapid screening of radionuclides is a concept currently being developed by Raddec International Ltd and is expected to lead to practical benefits. The basic principle is well established in other fields such as screening measurements of pH and chemical and biochemical species. The current approach incorporates an analyte-specific extractant that will interact with the target analyte (usually by adsorbing the analyte to a degree proportional to its concentration in the solution). The dip-stick can then be recovered for subsequent measurement. The dip-stick deployment and recovery can be readily achieved by non-specialists with minimal infrastructure or equipment. The dip-sticks can be designed to target specific analytes through selection of appropriate commercially available materials or purpose-designed extractants such as calixarenes/crown ethers, ion-imprinted polymers or metal organic frameworks. The approach is also ideal for the deployment of nano-particle extractants as the extractant is easily recoverable following deployment. The geometry of the dip-stick allows measurement using conventional liquid scintillation counting or using plate readers. Incorporation of scintillators, either integrated with the extractant or by using scintillating support materials, would permit further simplification of the detection systems. Dip-sticks can incorporate a single extractant to target one analyte (single extractant sticks—SES). Alternatively, multiple extractants can be incorporated in discrete zones (multiple extractant stick—MES), with measurement achieved using a spatially-resolved detector system to permit multi-radionuclide detection. The dip-stick technology does rely on the sample being in solution and some form of digestion is required for solid samples, although this can be simplified for screening purposes. It is therefore envisaged that the dip-stick technology could be applied to rapid screening of both aqueous and solid waste streams.

The aim of the current study was to evaluate the feasibility of using dip-stick technologies for rapid radionuclide screening in support of nuclear waste characterization. Two extractant systems were evaluated; (1) Sr-resin SES for ^90^Sr determination in acid leachates and (2) TEVA-resin SES for ^99^Tc determination in neutral solutions. Key operational parameters were assessed including the mass of resin loaded onto the dip-stick, the rate of analyte uptake and the correlation between activity on the dip-stick and activity concentration of the analyte in solution. The sensitivity of the dip-stick systems was compared with the operational limits associated with nuclear waste characterization.

## Theory

In principle, the quantity of radionuclide adsorbed by the dip-stick will be proportional to the activity concentration of the target radionuclide in solution, assuming that the quantity of the analyte is not approaching or exceeding the capacity of the adsorbent (Eq. 1).

1 $$A_{{({\text{s}},t)}} = b_{t} \left[ {A_{\text{aq}} } \right]_{0} ,$$

where *A*_(s,t)_ is the activity of the radionuclide on the dip-stick at time *t*, (Bq_s_). (*A*_aq_)_0_ is the initial activity concentration of the radionuclide in the test solution (Bq ml^−1^). *b*_*t*_ is the proportionality constant for time *t*, (Bq_s_ ml Bq_aq_^−1^).

The proportionality constant, *b*, is a function of a number of factors

2 $$b_{t} = f\left( {k_{eq,} {\raise0.7ex\hbox{${{\text{d}}k}$} \!\mathord{\left/ {\vphantom {{{\text{d}}k} {{\text{d}}t}}}\right.\kern-0pt} \!\lower0.7ex\hbox{${{\text{d}}t}$}},V,m_{{{\text{s}}, }} t} \right),$$

where *k*_eq_ is the effective distribution coefficient at equilibrium (ml g^−1^). d*k*/d*t* is the rate of change in effective distribution coefficient under non-equilibrium conditions (ml g^−1^ s^−1^). *V* is the volume of aqueous phase (ml). *m*_s_ is the mass of the extractant on the dip-stick (g). *t* is the exposure time, (s).

At any given time, *t*,

3 $$b_{t} = \frac{{\left( {m_{\text{s}} k_{t} } \right)}}{{1 + \frac{{\left( {m_{\text{s}} k_{t} } \right)}}{V}}},$$

where *k*_*t*_ is the effective distribution coefficient at time *t* (g ml^−1^).

## Experimental

**Fig. 1:**
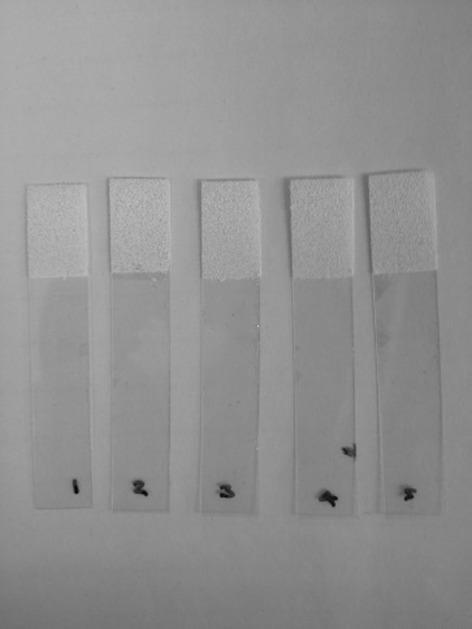
Example of SES dip-sticks (Sr-resin 100–150 µm)

Single extractant (SES) dip-sticks were prepared by coating the end of cellulose acetate strips with either Sr-resins or TEVA resin (Triskem, France). Only one side of the dip-stick was coated. Unless otherwise stated, 100–150 µm resins were used. Sr-resin sticks were also prepared using 50–100 µm Sr-resin to assess the impact of particle size on reaction rate. The active area of the strip was ~1 cm^2^ (Fig. 1). For commercial reasons, more detailed preparation procedures are not included.

To determine the mass of resin loaded onto the dip-stick (*m*_s_), ~0.5 g of TEVA resin was equilibrated with ~5000 Bq of ^99^Tc. The mixture was filtered and the Tc-loaded TEVA resin was dried and reweighed. Aliquots of the Tc-loaded TEVA resin were accurately weighed into a 22 mL polythene vial and 20 mL Gold Star scintillation cocktail was added. The ^99^Tc activity per gram of TEVA resin was then calculated. The Tc-loaded TEVA resin was then used to prepare a series of dip-sticks. Once prepared, the ^99^Tc activity on the dip-stick was measured using liquid scintillation counting and the mass of resin present on the dip-stick was calculated from the measured activity.

The rate of radionuclide uptake was assessed for Sr-SES and TEVA-SES dip-sticks. Five dip-sticks were prepared for each extractant using 100–150 µm resin. The effect of particle size on rate of uptake was also assessed for Sr-resin using dip-sticks prepared using 50–100 µm resin. Test solutions were prepared by spiking the target solution with known activities of ^90^Sr or ^99^Tc (NPL, UK). The dip-sticks were exposed to a test solution of either 8 M HNO_3_ (^90^Sr), or water at pH 7 (^99^Tc) containing a known activity concentration of the target radionuclide. The solutions were unstirred during exposure. The dip-stick was placed into the solution for a specified period of time, recovered and any adhering solution removed by quickly rinsing the test stick in a matrix-matched solution and drying with paper towel. The activity on the stick was measured by liquid scintillation counting. The rate of change in effective distribution coefficient (d*k*/d*t*) was then assessed.

The ratio of activity associated with the dip-stick to the initial activity concentration in solution (*b*_t_) was determined for Sr-SES and TEVA-SES dip-sticks. Dip-sticks were exposed to the test solutions containing the target radionuclide at a range of activity concentrations for a defined time. The activity adsorbed on the dip-stick was then measured and the correlation between the activity on the dip-stick and the activity concentration in solution was assessed. The passive adsorption of the radionuclide on an inert medium was also assessed by preparing a dip-stick using prefilter material (the support material used in the extractive resins) and exposing the ‘blank’ dip-stick to ^90^Sr solution. The limit of detection for each dip-stick was also calculated and compared with operational limits associated with nuclear waste characterization.

The activities of the radionuclides on the dip-stick and in the test solution were measured using Wallac 1220 Quantulus liquid scintillation counters. The dip-stick was transferred to a 22 mL polythene vial and 20 mL Gold Star scintillation cocktail (Meridian, UK) was added. Aliquots of test solution (typically 1 mL) were also sampled and mixed with 19 mL scintillation cocktail. Samples were typically counted along with a background sample for 3600 s.

## Results and discussion

### Mass of extractive-resin on dip-stick

**Fig. 2:**
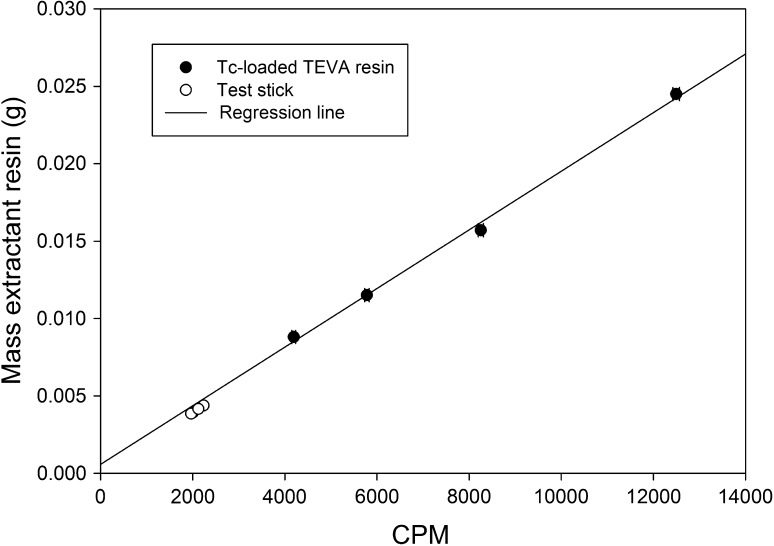
Correlation between ^99^Tc count rate and resin mass for Tc-loaded TEVA resin and Tc/TEVA-SES dip-sticks

Resin loading was initially assessed gravimetrically. Weighing of test sticks before and after loading of the extractive resin indicated that ~4 mg of Sr and TEVA (100–150 μm) resin were loaded onto an area of ~1 cm^2^. For the Sr-resin 50–100 μm, approximately 2 mg of resin could be loaded over the same area. However, this mass includes both the resin and the adhesive used to mount the resin, although the mass of the latter is considered to be low. To independently verify resin loading, TEVA resin, labeled with a known activity concentration of ^99^Tc was mounting onto five replicate sticks and the activity of ^99^Tc was measured by liquid scintillation counting. The measured value of 8.4 ± 0.6 kBq ^99^Tc per gram of loose TEVA resin agreed well with the theoretical maximum loading of 8.5 kBq per gram indicating near-quantitative uptake of the ^99^Tc on the resin. Direct measurement of increasing masses of the resin showed a linear relationship between activity and mass (Fig. 2). Measurement of the ^99^Tc activity associated with the Tc-TEVA resin loaded onto the TEVA-SES stick gave a mean resin loading of 0.0041 ± 0.0004 g (2*σ*; *n* = 5). This is in good agreement with the mass of Sr resin and TEVA resin loaded onto the test stick (100–150 μm) as determined gravimetrically.

### Rate of uptake on Sr, and TEVA-SES sticks

Replicate test sticks were exposed to a solution containing either ^90^Sr or ^99^Tc at a fixed concentration but for varying times (*t*_1_, *t*_2_…*t*_5_). The radionuclide activity on the test stick was measured and the effective distribution coefficient, *k*_t_ at time t, was determined according to Eq. (4)

4 $$k_{t} = \frac{{A_{\text{stick}} }}{{\left[ {A_{\text{aq}} } \right]_{t} \times m_{\text{s}} }}.$$

The effective distribution coefficient was then plotted against time and the data fitted to the first order exponential function (Eq. 4) using Sigmaplot™ regression analysis tool.

5 $$k_{t} = k_{\text{eq}} \left( {1 - e^{ - rt} } \right).$$

The coefficients of effective distribution coefficient at equilibrium, *k*_eq_, and the rate constant, *r* were then calculated from the fitted curve (Table 1).

**Table 1:** Uptake coefficients for rate of uptake

Radionuclide	Dip-stick	Particle size (μm)	*k*_eq_ (ml g^−1^)	Rate coefficient, *r* (s^−1^)
^90^Sr	Sr-SES	50–100	204	3.9 (8) × 10^−4^
^90^Sr	Sr-SES	100–150	169	2.0 (5) × 10^−4^
^99^Tc	TEVA-SES	100–150	280	1.5 (5) × 10^−4^

**Fig. 3:**
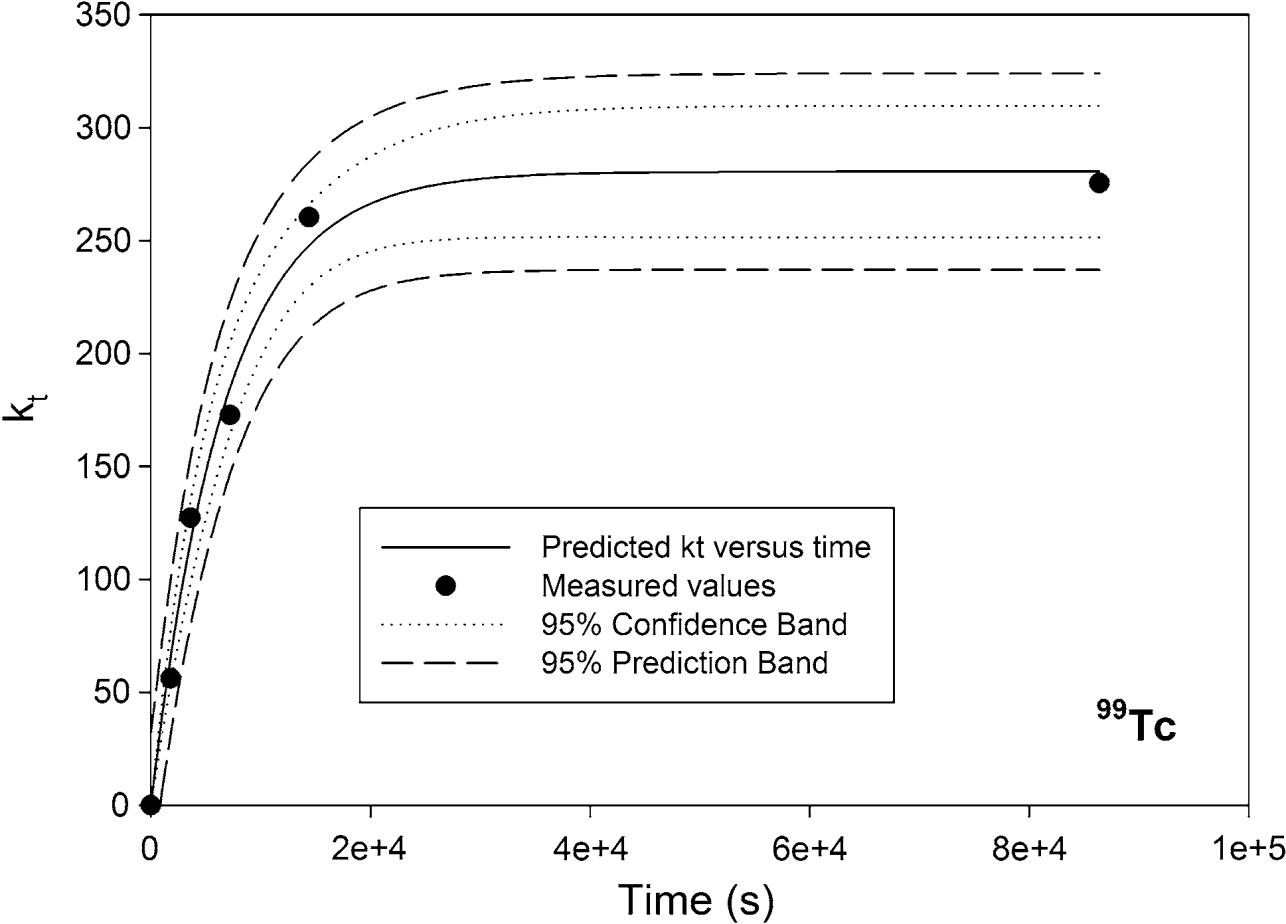
Rate of ^99^Tc uptake on TEVA-SES stick

**Fig. 4:**
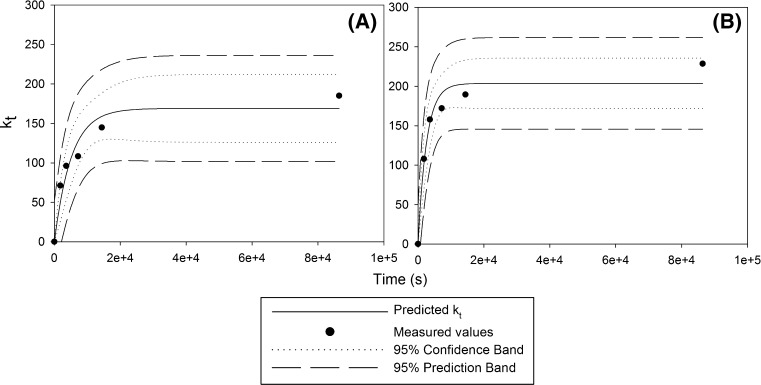
Rate of ^90^Sr uptake on Sr-SES stick (**a** 100–150 μm; **b** 50–100 μm)

As a first approximation, the experimentally-determined uptake data were described reasonably well by the first order equation (Figs. 3, 4) with regression coefficients values ranging from 0.93 to 0.99. The rate coefficient, *r*, was comparable for the 100–150 μm Sr-SES and TEVA-SES stick. The rate coefficient for the 50–100 μm Sr-SES stick was aprpopximately two times the coefficient for the 100–150 μm stick. The highest *k*_eq_ value was observed for the TEVA-SES stick. For the Sr-SES sticks, the 50–100 μm stick gave a higher *k*_eq_ value compared with the 100–150 μm stick (Table 1).

### Response of dip-stick to varying aqueous activity concentration

**Fig. 5:**
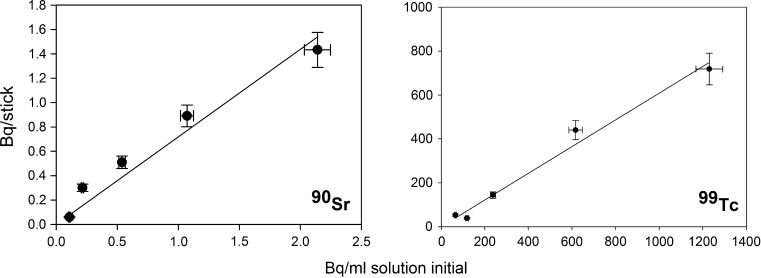
Activity on dip-stick versus activity concentration in solution for (*left*) ^90^Sr on Sr-SES stick (exposure time 1140 min) and (*right*) ^99^Tc on TEVA-SES stick (exposure time 300 min)

For both the Sr-SES and the TEVA-SES dip-sticks, the measured activity associated with the dip-stick after deployment was proportional to the activity concentration of the radionuclide in solution (Fig. 5). Such a relationship is expected assuming that the capacity of the resin has not been exceeded. The calculated proportionality constant, *b*_t_, for the Sr-SES dip-stick was 0.72 Bq_s_ ml Bq_aq_^−1^ (*t* = 68400 s; *r* ^2^ = 0.946), whereas the value for the TEVA-SES dip-stick was 0.61 Bq_s_ ml Bq_aq_^−1^ (*t* = 1800 s; *r* ^2^ = 0.982). These compare with b_t_ values calculated from the rate experiments of 0.6 Bq_s_ ml Bq_aq_^−1^ (Sr-SES) and 0.8 Bq_s_ ml Bq_aq_^−1^ (TEVA-SES). The similarity of the two measured coefficients is consistent with the calculated k_eq_ values but is unexpected given the difference in published distribution coefficients (D_w_) for the two resins (~200 for Sr resin versus 10^4^ for TEVA resin, [6, 7]). This implies that radionuclide partitioning between the aqueous and solid phase is not thermodynamically controlled (particularly as the systems were unmixed). The activity measured in the ‘blank’ dip-stick (comprising inert prefilter material only) was <0.02 Bq/stick per Bq/mL solution confirming that uptake of the radionuclide on the Sr-SES dip-stick involved active interaction with the extractive resin and not passive physisorption to the dip-stick.

The sensitivity of the dip-stick system will depend on the deployment time and count time. It is envisaged that the deployment time will be fixed and specified for a given target sensitivity. For a 240 min deployment and 60 min count time, the limit of detection is <0.1 Bq/mL of solution. For solid samples, this would equate to 1 Bq/g assuming that 0.5 g of solid sample is leached with 5 mL of reagent. This detection limit compares favourably with the out-of-scope limits of 1 Bq/g for ^90^Sr and ^99^Tc [8]. Further developments will be aimed at reducing the exposure time to permit more rapid deployment. The sensitivity of the system could be further improved by increasing the active surface area of the dip-stick or by increasing counting times. It is also noted that consideration must be given to the effect of interfering radionuclides on test stick response. For example, the Sr-SES stick would be expected to respond to ^210^Pb in solution [9]. Likewise, tetra-valent actinides would co-extract with TcO_4_^−^ on the TEVA-SES resin [7]. The use of multiple extractant zones would assist in deconvolving the signal from multiple radionuclides and barrier films could be used to prevent interfering radionuclides from interacting with the extractant. In addition, the observed response may be impacted by the presence of certain matrix elements in solution which could compete for adsorption sites or suppress uptake of the target analyte. These will be evaluated in future research.

## Conclusions

The concept of the dip-tick for the assessment of radionuclide concentrations in aqueous solution has been successfully demonstrated using Sr-resin and TEVA-resin based dip-sticks. The quantity of radionuclide (either ^90^Sr or ^99^Tc) adsorbed onto the active area of the stick is proportional to the initial activity concentration present in solution. Further research is now required to assess the impact of competing ions on the performance of the test stick and to evaluate the effects of geometry, surface area and uptake kinetics and to optimize the operation of the test stick system accounting for these factors. The applicability of the test stick approach will be further extended through the development and evaluation of other extractant systems and to the application of multiple-extractant sticks measured using plate reader technologies. Finally, the adoption of these systems will require further development of simplified sample preparation procedures and the testing of the systems using operationally-exposed samples. However, it is envisaged that the successful development of such a testing approach could offer significant advantages for waste sentencing and facilitate more rapid screening at the point of waste production.
